# Validação de um Algoritmo de Inteligência Artificial para a Predição Diagnóstica de Doença Coronariana: Comparação com um Modelo Estatístico Tradicional

**DOI:** 10.36660/abc.20200302

**Published:** 2021-11-22

**Authors:** Luis Correia, Daniel Lopes, João Vítor Porto, Yasmin F. Lacerda, Vitor C. A. Correia, Gabriela O. Bagano, Bruna S. B. Pontes, Milton Henrique Vitoria de Melo, Thomaz E. A. Silva, André C Meireles

**Affiliations:** 1 Escola Bahiana de Medicina e Saúde Pública Salvador BA Brasil Escola Bahiana de Medicina e Saúde Pública, Salvador, BA – Brasil; 2 Hospital São Rafael Salvador BA Brasil Hospital São Rafael, Salvador, BA – Brasil

**Keywords:** Estudos de Validação, Inteligência Artificial, Doença da Artéria Coronariana/diagnóstico, Interpretação Estatística de Dados

## Abstract

**Fundamento:**

A análise prognóstica multivariada tem sido realizada tradicionalmente por modelos de regressão. No entanto, muitos algoritmos surgiram, capazes de traduzir uma infinidade de padrões em probabilidades. A acurácia dos modelos de inteligência artificial em comparação à de modelos estatísticos tradicionais não foi estabelecida na área médica.

**Objetivo:**

Testar a inteligência artificial como um algoritmo preciso na predição de doença coronariana no cenário de dor torácica aguda, e avaliar se seu desempenho é superior a do modelo estatístico tradicional.

**Métodos:**

Foi analisada uma amostra consecutiva de 962 pacientes admitidos com dor torácica. Dois modelos probabilísticos de doença coronariana foram construídos com os primeiros 2/3 dos pacientes: um algoritmo *machine learning* e um modelo logístico tradicional. O desempenho dessas duas estratégias preditivas foi avaliado no último terço de pacientes. O modelo final de regressão logística foi construído somente com variáveis significativas a um nível de significância de 5%.

**Resultados:**

A amostra de treinamento tinha idade média de 59 ± 15 anos, 58% do sexo masculino, e uma prevalência de doença coronariana de 52%. O modelo logístico foi composto de nove preditores independentes. O algoritmo *machine learning* foi composto por todos os candidatos a preditores. Na amostra teste, a área sob a curva ROC para predição de doença coronariana foi de 0,81 (IC95% = 0,77 – 0,86) para o algoritmo *machine learning*, similar à obtida no modelo logístico (0,82; IC95% = 0,77 – 0,87), p = 0,68.

**Conclusão:**

O presente estudo sugere que um modelo *machine learning* acurado não garante superioridade à um modelo estatístico tradicional

## Introdução

Nas últimas décadas, a capacidade dos computadores em gerar e armazenar dados melhorou substancialmente, produzindo grandes bancos de dados, de alta complexidade. A modelagem estatística tradicional tem a vantagem de ser simples, uma vez que ela ajusta a relação entre preditores de desfechos em uma fórmula de regressão. No entanto, esses modelos possuem algumas premissas difíceis de serem cumpridas em conjuntos complexos de informações: número limitado de variáveis, distribuição adequada, independência nas observações, ausência de multicolinearidade, e problemas de interações. Por outro lado, o mecanismo de predição da inteligência artificial é baseado em algoritmo, sem premissas ou limite de variáveis. Assim, diferente da modelagem estatística, algoritmos preditivos não se tornam menos precisos à medida que os dados se tornam mais complexos. Nos cenários de “big data”, a inteligência artificial torna-se mais precisa que a estatística tradicional.^1,2^

Informações médicas podem apresentar vieses caso não sejam coletadas por meio de protocolos pré-estabelecidos. Por isso, a abordagem epidemiológica tradicional, de pequenos conjuntos de dados, coletados prospectivamente, é a escolha mais apropriada na pesquisa médica.^3^ Portanto, é importante investigar se a inteligência artificial continua superior à modelagem estatística se exposta a amostras de tamanho moderado e número limitado de variáveis, como nos estudos epidemiológicos.

A predição de Doença Arterial Coronariana (DAC) em pacientes com dor torácica aguda é um grande desafio para o médico de emergência, quem tem que decidir entre a alta do paciente, a realização de outros testes não invasivos, ou a opção direta por angiografia invasiva. Dar alta a um paciente com doença coronariana instável pode ter efeitos devastadores, por outro lado, admitir qualquer pessoa com dor torácica pode ter consequências não intencionais.^4^ Nesse processo, a probabilidade de DAC obstrutiva deve conduzir a tomada de decisão médica.^5^

No presente estudo, utilizamos dados de um registro prospectivo de dor torácica para construir um modelo de *machine learning* para predizer doença coronariana obstrutiva. Nosso objetivo foi avaliar se um algoritmo de inteligência artificial é um melhor preditor que a regressão logística em um conjunto tradicional de dados epidemiológicos simples, considerando tanto propriedades discriminatórias como de calibração.

## Métodos

### Seleção da amostra

De setembro de 2011 a novembro de 2017, todos os pacientes admitidos por dor torácica e suspeita clínica de DAC (independentemente de resultados de eletrocardiograma ou níveis de troponina) na unidade coronariana de nosso hospital foram incluídos no estudo. O critério de exclusão foi a recusa do paciente em participar. Conforme definido *a priori*, um total de 962 pacientes foram divididos em amostra de derivação (primeiros dois terços, n= 641) ou amostra de validação (último terço, n= 321). O estudo foi aprovado pelo comitê de ética da instituição e consentimento informado foi obtido dos participantes.

#### Preditores de DAC obstrutiva

Na admissão (basal), três conjuntos de variáveis foram registrados como candidatos à predição de DAC obstrutiva. Primeiro, 13 variáveis relacionadas à história médica e apresentação clínica; segundo, 14 características de desconforto torácico; terceiro, 11 variáveis relacionadas a achados anormais em testes de imagem ou laboratoriais na admissão: alterações isquêmicas no eletrocardiograma (inversão da onda T ≥ 1 mm ou desvio dinâmico do segmento ST ≥ 0,5 mm), troponina positiva (> percentil 99 para a população geral; Ortho-Clinical Diagnostics, Rochester, NY, EUA), N-terminal do peptídeo natriurético tipo B (NT-ProBNP, teste de imunoensaio fluorescente, Biomérieux, França), proteína C-reativa ultrassensível (CRP, nefelometria, Dade-Behring, EUA), d-dímero (ensaio imunoenzimático, Biomérieux, França), lipoproteína de baixa densidade (LDL cholesterol; equação de Friedwald), creatinina, contagem de leucócitos, glicemia, e hemoglobina. As dosagens laboratoriais foram realizadas a partir de plasma coletado no momento de chegada no departamento de emergência. História médica e características de dor torácica foram registradas por três investigadores (M.C., A.M.C., R.B.) treinados para entrevistar os pacientes de maneira padronizada, a fim de minimizar a ocorrência de viés e melhorar a reprodutibilidade. Sinais radiológicos de insuficiência ventricular e eletrocardiograma foram interpretados pelo mesmo investigador (L.C.).

### Desfechos

O desfecho primário a ser predito pelo modelo foi diagnóstico de DAC obstrutiva, definida por testes subsequentes realizados durante a internação hospitalar. Os dados relacionados ao desfecho foram coletados por três investigadores (M.C., A.M.C., R.B.), e confirmados por um quarto investigador (L.C.). Para a avaliação diagnóstica, os pacientes foram submetidos à angiografia coronária invasiva ou a um teste provocativo (ressonância magnética de perfusão, tomografia computadorizada por emissão de fóton único, ou ecocardiografia sob estresse com dobutamina), a critério do cardiologista assistente. Em caso de um teste não-invasivo positivo, a DAC obstrutiva foi definida como presença de estenose ≥ 70% por angiografia. Um teste não-invasivo normal indicou ausência de DAC obstrutiva, e nenhum outro exame foi necessário. Independentemente dos testes coronários, os pacientes foram classificados como “DAC não obstrutiva”, se uma das seguintes condições fosse diagnosticada por exame de imagem – pericardite, embolismo pulmonar, disseção córtica, ou pneumonia.

### Análise estatística

O teste de Shapiro-Wilk foi usado para testar se os dados apresentavam distribuição normal. Para análise descritiva, utilizamos média e desvio padrão para variáveis contínuas com distribuição normal, e mediana e intervalo interquartil para as variáveis contínuas sem distribuição normal. As variáveis categóricas foram descritas como frequências. Na amostra de derivação, utilizamos primeiramente o teste t de Student não pareado para as variáveis contínuas com distribuição normal e o teste do qui-quadrado para análise univariada das variáveis categóricas. As variáveis numéricas sem distribuição normal foram analisadas pelo teste de Mann-Whitney não paramétrico. Em seguida, as variáveis com p<0,20 na análise univariada foram incluídas na análise de regressão logística multivariada para predição de DAC obstrutiva.

Modelos multivariados foram desenvolvidos pelo método *stepwise*. Todas as variáveis foram ajustadas (entrada forçada) em um modelo de regressão logística e, em cada etapa, a variável menos significativa foi removida do modelo pelo teste de Wald. Inicialmente, foram construídos três modelos intermediários, de acordo com o tipo das variáveis preditivas (história médica, características da dor torácica ou exame físico/testes laboratoriais). Preditores independentes (p<0,10) em cada modelo intermediário foram incluídos como covariáveis no modelo final, construído incluindo-se somente variáveis significativas, com nível de significância a 5%.

O algoritmo *machine learning* reconhece padrões em características clínicas associados com probabilidades de desfecho, A análise discriminatória de Fisher foi usada para a criação de dendrogramas, os quais foram combinados repetidamente até a taxa de erros indicar um ótimo desempenho. A amostra de derivação foi usada para construir o algoritmo *machine learning*. Diferente da regressão logística, não houve uma pré-seleção de variáveis e todos os 55 parâmetros foram incluídos sem nenhuma eliminação. A influência de cada variável no cálculo de probabilidade foi definida pela pureza dos nós e a porcentagem de aumento do erro associado. Como resultado da análise gráfica, realizamos 8000 interações de combinações.

Os dois modelos foram comparados na amostra de validação. Áreas sob curvas ROC (Característica de Operação do Receptor) foram usadas para testar discriminação, e comparadas entre os modelos pelo teste de DeLong. A calibração foi testada pelo teste de Hosmer-Lemeshow (aplicado nas probabilidades geradas pelos modelos), e calculando-se a inclinação e o intercepto da reta de regressão probabilidade predita média *versus* incidência de eventos, observada por decis de predição (um modelo perfeitamente calibrado tem um intercepto de 0 e uma inclinação de 1). Antes de realizar a regressão linear, as seguintes premissas foram verificadas: relação linear, independência das observações, normalidade dos resíduos, homoscedasticidade dos resíduos.

A significância estatística foi definida como p < 0,05. O programa SPSS foi usado para a análise dos dados.

### Determinação do tamanho amostral

O *machine learning* não possui premissas quanto ao tamanho da amostra. Para a regressão logística, o conjunto de derivação foi planejado para permitir a inclusão de pelo menos 15 covariáveis no modelo de regressão logística. O cálculo foi realizado com base nas seguintes premissas: prevalência de DAC de 50% e necessidade de 10 eventos para cada covariável no modelo de regressão logística.^6,7^ Portanto, seria necessário um mínimo de 300 pacientes na amostra de derivação. A amostra de validação foi estabelecida para testar a acurácia de discriminação pela análise da curva ROC. Partindo-se da premissa de uma AUC de 0,70, para um poder de 90% de se rejeitar a hipótese nula de uma AUC de 0,50, e alfa de 5%, seria necessário um mínimo de 85 pacientes. Portanto, um mínimo de 100 pacientes seria preciso no grupo de validação. Tais premissas foram cumpridas. A análise dessa única amostra foi realizada e concluída em janeiro de 2018 para evitar a realização de múltiplas análises.

## Resultados

### Características da amostra de derivação

Foram estudados 641 pacientes, com idade de 59 ± 15 anos, 58% homens, 30% com história prévia de doença coronariana. O tempo mediano entre o início dos sintomas e a primeira avaliação clínica no hospital foi de 4,2 horas (intervalo interquartil 1,9-14 horas). Utilizando o protocolo do estudo, identificamos 330 pacientes com DAC obstrutiva, uma prevalência de 52%. Todos esses casos tiveram o diagnóstico confirmado por angiografia coronária invasiva. Em relação aos 311 pacientes sem DAC, 93 foram classificados por uma angiografia negativa, 169 por um teste não-invasivo negativo, e 52 apresentaram outro diagnóstico dominante (14 embolia pulmonar, cinco dissecção da aorta, 28 pericardite, dois pneumonia).

### Características da amostra de validação

Foram estudados 221 pacientes, com algumas características similares ao do grupo de derivação – idade de 59 ± 16 anos, 58% do sexo masculino, 22% com história de doença coronariana. O tempo mediano entre o início dos sintomas e a primeira avaliação clínica no hospital foi de 7,0 horas (intervalo interquartil 2,4-23 horas). Utilizando o protocolo do estudo, identificamos 163 pacientes com DAC obstrutiva, uma prevalência de 51%. Todos esses casos tiveram o diagnóstico confirmado por angiografia coronária invasiva. Dos 158 pacientes sem DAC, 88 foram classificados por uma angiografia negativa, 13 por um teste não-invasivo negativo, e 57 apresentaram outro diagnóstico dominante (12 embolia pulmonar, dois dissecção da aorta, 25 pericardite, cinco pneumonia).

### Desenvolvimento do modelo logístico

Entre as 13 variáveis relacionadas à história médica de apresentação clínica, sete estavam associados positivamente com DAC obstrutiva (p<10%): idade, sexo masculino, disfunção do ventrículo esquerdo aguda, história prévia de DAC, diabetes, tabagismo, e sintomas desencadeados por exercício – Tabela 1. Quando essas sete variáveis foram incluídas na regressão logística, história prévia de DAC perdeu significância, e todas as demais mantiveram o nível significativo de < 5% (modelo intermediário 1, Tabela 2).

**Tabela 1:** Comparação da história médica, características da dor torácica, e exames laboratoriais entre pacientes com e sem doença arterial coronariana obstrutiva da amostra de derivação

	DAC obstrutiva		Valor de p
	Sim (N = 330)	Não (N = 311)	
***História médica***			
Idade (anos)	63 ± 13	56 ± 16	< 0,001
Sexo masculino	226 (69%)	148 (48%)	< 0,001
Índice de massa corporal (Kg/m^2^)	28 ± 4,8	28 ± 5,9	0,86
Pressão arterial sistólica (mmHg)	154 ± 32	152 ± 30	0,55
Frequência cardíaca (bpm)	78,7 ± 19	79,4 ± 19	0,63
Raio X e sinais clínicos de IVE	41 (13%)	6 (2,0%)	< 0,001
História de DAC	113 (34%)	77 (25%)	0,01
Diabetes	122 (37%)	74 (24%)	< 0,001
Hipertensão sistêmica	236 (72%)	210 (68%)	0,27
Tabagismo atual	44 (13%)	26 (8,4%)	0,04
História familiar de DAC	87 (26%)	79 (25%)	0,78
Indução por exercício	50 (15%)	22 (7,1%)	0,001
Indução emocional	8 (2,4%)	15 (4.8%)	0,10
***Características da dor torácica***			
Lado anterior esquerdo (local)	268 (81%)	261 (84%)	0,37
Dor opressiva	189 (57%)	157 (51%)	0,09
Irradiação para o pescoço	82 (25%)	74 (24%)	0,76
Irradiação para o braço esquerdo	120 (36%)	93 (30%)	0,08
Sintomas vagais	146 (44%)	132 (42%)	0,65
Intensidade grave	185 (56%)	150 (48%)	0,05
Número de episódios	1 (1 – 2)	1 (1 – 3)	0,14
Duração (minutos)	75 (20 – 129)	60 (11 – 214)	0,07
Intensidade (escala 1 – 10)	7,7 ± 2.4	7,3 ± 2,4	0,03
Alívio com nitratos	134 (41%)	98 (32%)	0,02
Similar a infarto prévio	105 (32%)	76 (24%)	0,04
Piora com compressão	19 (5,8%)	43 (14%)	0,001
Piora com mudança de posição	53 (16%)	60 (19%)	0,28
Piora com movimento do braço	19 (5,8%)	31 (10%)	0,05
Piora com inspiração profunda	34 (10%)	68 (22%)	< 0,001
***Exames laboratoriais na admissão***			
Alterações isquêmicas no ECG	219 (66%)	119 (38%)	< 0,001
Troponina positiva	215 (65%)	102 (33%)	< 0,001
NT-proBNP (pg/mL)	432 (155 – 1212)	73 (24 – 301)	< 0,001
Creatinina plasmática (mg/dL)	0,90 (0,80 – 1,20)	0,80 (0,70 – 1,1)	< 0,001
LDL-colesterol (mg/dL)	104 ± 53	108 ± 74	0,46
Glicose plasmática (mg/dL)	130 (99 – 160)	107 (90 – 145)	0,009
Proteína C-reativa (mg/L)	7,4 (2,4 – 15)	6,3 (1,6 – 15)	0,003
Contagem de leucócitos	7,600 (6,050 – 10,100)	7,200 (5,700 – 9,550)	0,04
Plaquetas	232 (192 – 290)	232 (197 – 274)	0,83
D-dímero (ng/mL)	474 (279 – 981)	424 (278 – 913)	0,43
Hemoglobina (g/dL)	14,1 ± 1,9	13,7 ± 1,7	0,11

DAC: doença arterial coronariana; história familiar de DAC implica parente de primeiro grau do sexo feminino com a doença em idade inferior a 55 anos, ou do sexo masculino com a doença em idade inferior a 45 anos; IVE: insuficiência ventricular esquerda; NT-pro-BNP: N-terminal do peptídeo natriurético tipo B; ECG: eletrocardiograma; LDL: lipoproteína de baixa densidade.

**Tabela 2:** Modelos de regressão logística intermediária de história médica (Modelo 1), características de dor torácica (Modelo 2), e testes laboratoriais (Modelo 3)

Variáveis	Nível de significância multivariada
***Modelo 1 (história médica)***	
Idade (anos)	< 0,001
Sexo masculino	< 0,001
Raio X ou sinais clínicos de IVE	< 0,001
Indução por exercício	0,005
Diabetes	0,009
Tabagismo	0,02
DAC prévia	0,32
***Modelo 2 (características da dor torácica)***	
Piora com inspiração profunda	0,001
Piora com compressão	0,01
Intensidade grave	0,01
Dor opressiva	0,06
Similar a infarto prévio	0,08
Irradiação para o braço esquerdo	0,16
Alívio com nitrato	0,25
Duração (minutos)	0,32
Piora com movimento do braço	0,67
***Modelo 3 (exames laboratoriais)***	
Alterações isquêmicas no ECG	< 0,001
Troponina positiva	< 0,001
NT-proBNP (pg/mL)	0,89
Creatinina plasmática (mg/dL)	0,17
Glicose plasmática (mg/dL)	0,12
Proteína C-reativa (mg/L)	0,58
Contagem de leucócitos	0,80

DAC: doença arterial coronariana; IVE: insuficiência ventricular esquerda; NT-pro-BNP: N-terminal do peptídeo natriurético tipo B; ECG: eletrocardiograma.

Em relação às características da dor torácica, entre 14 variáveis, seis apresentaram associação com DAC: dor opressiva, irradiação para o braço esquerdo, intensidade grave, duração em minutos, alívio com o uso de nitrato, similaridade com infarto prévio; e três apresentaram associação negativa com DAC: piora com compressão, movimento dos braços e inspiração profunda (Tabela 1). Quando essas nove variáveis foram adicionadas à regressão logística, somente três permaneceram significativos a um nível <5% - piora com compressão, inspiração profunda, e intensidade grave (modelo intermediário 2, Tabela 2).

Dos 11 exames laboratoriais, sete estavam positivamente associados com DAC: isquemia no eletrocardiograma, troponina positiva, creatinina, glicemia, NT-pro-BNP, PCR, contagem de leucócitos (Tabela 1). Quando essas variáveis foram incluídas na regressão logística, somente isquemia no eletrocardiograma e troponina positiva permaneceram significativos no nível de p<0,05 (modelo intermediário 3, Tabela 2).

As 11 variáveis significativas no modelo intermediário foram incluídas na análise final de regressão logística, gerando um modelo final com nove variáveis significativas para predizer a presença de DAC: idade, sexo masculino, isquemia no eletrocardiograma, troponina positiva, disfunção do ventrículo esquerdo, indução por exercício, tabagismo, diabetes, e piora com inspiração profunda como a única “variável protetora”. Coeficientes de regressão e *odds ratios* estão descritos na Tabela 3.

**Tabela 3:** Modelo de regressão logística final definindo os preditores independentes de doença arterial coronariana

Variáveis	beta	Odds ratio (95% IC)	Valor de p
Idade (cada ano)	0,032	1,03 (1,02 – 1,05)	< 0,001
Sexo masculino	1,04	2,8 (1,9 – 4,2)	< 0,001
Alterações isquêmicas no ECG	1,05	3,0 (1,96 – 4,2)	< 0,001
Troponina positiva	1,03	2,8 (1,9 – 4,1)	< 0,001
Sinais de IVE	1,49	4,4 (1,7 – 12)	0,002
Indução por exercício	0,93	2,5 (1,4 – 4,7)	0,003
Tabagismo	0,63	1,9 (1,5 – 3,4)	0,03
Diabetes	0,53	1,7 (1,1 – 2,6)	0,01
Piora com inspiração profunda	- 0,93	0,39 (0,23 – 0,68)	0,001
*Constante*	-3,70	----	----
***Variáveis excluídas***			
Intensidade grave	----	----	0,06
Piora com compressão	----	----	0,20

Teste de Hosmer-Lemeshow = 4,1; p = 0,85. Área sob a curva ROC do modelo = 0,81; IC95% = 0,77 – 0,84; p < 0,001. ECG: eletrocardiograma; IVE: insuficiência ventricular esquerda.

### Desenvolvimento do modelo *machine learning*

Todas as 55 variáveis relacionadas história médica, apresentação clínica, características da dor torácica e exames laboratoriais foram incluídas no modelo *machine learning*. O desempenho de cada variável no modelo está apresentado na Tabela 4 por parâmetros de pureza dos nós e porcentagem de aumento no erro associado.

**Tabela 4:** Modelo de *machine learning* mostrando o peso de cada variável em definir probabilidade, de acordo com os parâmetros de pureza dos nós e porcentagem de aumento do erro associado

	Parâmetros	
	Pureza dos nós	Aumento do erro (%)
Idade (anos)	9,966665	0,015613620
Sexo masculino	2,8464500	0,007500700
Peso (kg)	4,1309610	0,001209398
Altura (cm)	3,4111841	0,001045826
Pressão arterial sistólica (mmHg)	4,9687120	0,001186313
Pressão arterial diastólica (mmHg)	3,8970542	0,000573540
Frequência cardíaca (bpm)	4,8355910	0,001049536
Raio X ou sinais clínicos de IVE	1,5479285	0,002145387
História de DAC	0,774541	0,000883823
História de angioplastia	0,809141	0,000852728
Revascularização cirúrgica prévia	0,407289	0,000246474
História de infarto	0,502479	0,000155925
Doença carotídea	0,352677	0,000111797
Doença arterial periférica	0,237674	0,000046758
Diabetes	0,606332	0,00041533
Hipertensão sistêmica	0,680378	0,00059024
Tabagismo atual	0,515775	0,00027025
História familiar de DAC	0,471644	0,00002877
Terapia com estatina	0,496937	0,00023743
Terapia com aspirina	1,004764	0,00120421
Insuficiência renal crônica	0,137357	-0,000055424
Diálise	0,016785	0,000007401
Menopausa	0,683362	0,00094085
Terapia de reposição hormonal	0,379223	0,00010860
Indução física /emocional	1,951236	0,00097193
Lado esquerdo anterior (local)	0,42644	0,00011250
Dor opressiva	0,90551	0,00070792
Irradiação para o pescoço	0,41147	-0,00011320
Irradiação para o braço esquerdo	0,70464	0,00025748
Sintomas vagais	0,493875	0,00003483
Intensidade grave	0,624608	0,00016137
Intensidade (0 – 10)	0,696121	0,00053586
Número de episódios	1,701348	0,00006361
Duração (minutos)	0,493875	0,00089453
Intensidade (escala 1-10)	2,604802	0,00053586
Alívio com nitratos	4,880035	0,00140420
Similar a infarto prévio	0,696121	0,000699946
Piora com compressão	0,905519	0,000707922
Piora com mudança de posição	0,384833	0,000041857
Piora com movimento do braço	0,295489	-0,000075263
Piora com inspiração profunda	1,006767	0,000973174
Alterações isquêmicas no ECG	4,880035	0,009409961
Troponina positiva	7,935190	0,002336380
NT-proBNP (pg/mL)	17,39237	0,00367361
Creatinina plasmática (mg/dL)	4,497093	0,00040330
Colesterol total (mg/dL)	4,291174	0,00298651
LDL-colesterol (mg/dL)	4,246389	0,00159658
HDL-colesterol (mg/dl)	6,131821	0,00596194
Triglicerídeos (mg/dl)	5,213428	0,00397991
Glicose plasmática (mg/dl)	4,115463	0,00222222
Proteína C reativa (mg/L)	3,948830	0,00315613
D-dímero	3,418193	-0,00010837
Contagem de leucócitos	4,7122731	0,00034806
Hemoglobina (g/dl)	6,0717680	0,00230890
Plaquetas	5,0908595	0,00103027

DAC: doença arterial coronariana; IVE: insuficiência ventricular esquerda; NT-proBNP: terminal do peptídeo natriurético tipo B; ECG: eletrocardiograma; LDL: lipoproteína de baixa densidade; HDL: lipoproteína de alta densidade.

### *Machine learning* versus regressão logística (amostra de validação)

**Figura 1:**
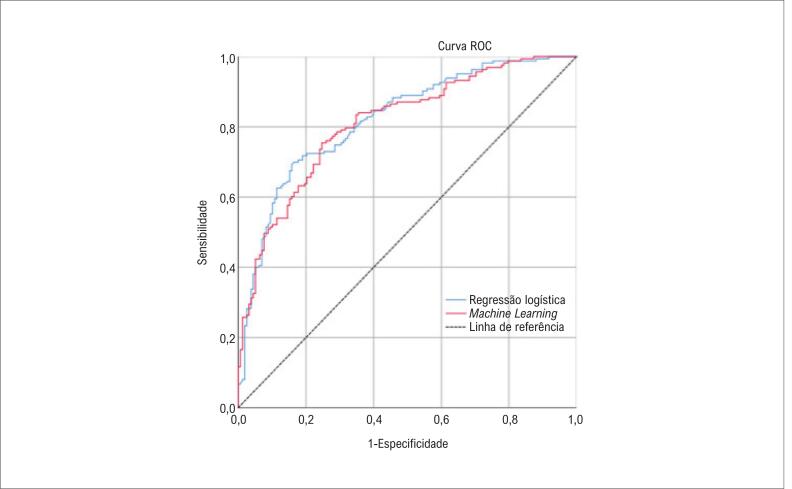
Área sob as curvas ROC do modelo de aprendizado de máquina e modelo de regressão logística, respectivamente 0,81 (IC 95% = 0,77 - 0,86) e 0,82 (IC 95% = 0,78 - 0,87).

Em relação à discriminação, a área sob a curva ROC das probabilidades foi de 0,81 (IC95% = 0,77 – 0,86), muito semelhante à área sob a curva do modelo de regressão logística de 0.82 (IC95% = 0,78 – 0,87), p = 0,68 (Figura 1).

Em relação à calibração, embora ambos os modelos tenham sido validados pelo teste de Hosmer-Lemeshow, o modelo logístico apresentou um nível de significância mais baixo da diferença entre os valores preditos e observados (qui-quadrado = 6,2; p = 0,62), quando comparado ao *machine learning* (qui-quadrado = 12,9; p = 0,11), o que sugere uma melhor calibração do primeiro modelo.

**Figura 2:**
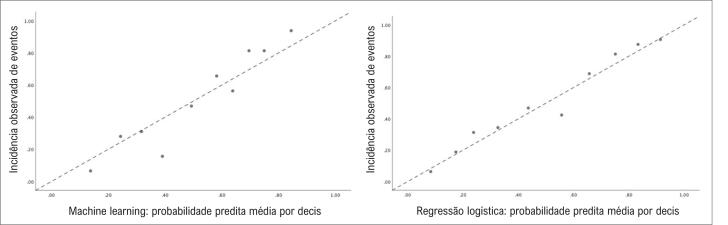
Gráfico de dispersão para análises de regressão linear entre os valores preditivos médios por decis e as incidências observadas. A figura A indica a calibração do modelo de aprendizado de máquina (interceptação = -0,119, inclinação = 1,228, r = 0,953). O painel B mostra a calibração do modelo de regressão logística (interceptação = 0,010, inclinação = 1,004, r = 0,981).

Esses dados foram reforçados pelo fato de que a regressão linear entre a probabilidade predita e a incidência observada de eventos por decis de predição mostrou um intercepto de 0,010 (IC95% = -0,083 – 0,103) e inclinação de 1,004 (IC95% = 0,840 – 0,168) para regressão logística (r = 0,981). Para o *machine learning*, foram observados intercepto de -0,119 (IC95% = -0,296 – 0,059) e inclinação de 1,228 (IC95% 0,909 – 1,547; r = 0,953) (Figura 2).

## Discussão

No presente estudo, testamos o conceito de se construir um instrumento *machine learning* para a predição de DAC em uma pequena amostra de pacientes com dor torácica aguda na admissão, com base em dados epidemiológicos coletados prospectivamente, e um número pequeno de variáveis. Primeiro, confirmamos que a inteligência artificial pode ser construída a partir desse tipo de dados e ser preciso na discriminação (sim ou não) e na calibração (predição de probabilidade); segundo, nossa análise de validação sugeriu que a inteligência artificial não é superior à estatística tradicional nessas circunstâncias.

Nos anos 50, o psicólogo Paul Meehl mostrou que a predição estatística é geralmente superior à predição clínica realizada pelo julgamento humano.^8^ Essa ideia foi reforçada pelo trabalho de Daniel Kahneman, ganhador do prêmio Nobel, que descreveu uma gama de vieses cognitivos responsáveis por imprecisão no método heurístico.^9^ Tais conceitos deram suporte a ênfase de se utilizar modelos estatísticos como a melhor abordagem baseada em evidências para predições diagnósticas e prognósticas. Mais recentemente, a inteligência artificial surge como uma técnica mais robusta de se construir instrumentos preditivos.

Tipicamente, a inteligência artificial deriva-se de grandes bancos de dados, disponíveis de registros eletrônicos ou interfaces de rede.^10^ Ela provê acurácia devido ao enorme tamanho amostral, e ausência de premissas em relação ao número de variáveis, distribuição, independências das observações, multicolinearidade e questões de interações.^1^ Contudo, uma vez que esses grandes conjuntos de dados não são coletados para propósitos científicos, faltam-lhes qualidade da informação.^3^ Por outro lado, estudos prospectivos epidemiológicos, com coleta de dados padronizada, planejada, e *a priori*, são os melhores métodos para a geração de conjuntos de dados de qualidade ideal. Nessas circunstâncias, as modelagens estatísticas tradicionais geralmente têm suas premissas cumpridas e apresentam um bom desempenho. Portanto, a questão é: nessas circunstâncias ideais de modelagem estatística, a inteligência artificial ainda é uma técnica superior?

No cenário de síndromes coronárias agudas e conjuntos de dados tradicionais, quatro autores comparam o *machine learning* com a estatística. Três dos estudos avaliaram o prognóstico na síndrome coronária aguda e compararam o *machine learning* com escores de risco, mostrando certa superioridade da inteligência artificial quanto à capacidade de discriminação.^11-13^ No entanto, nesses estudos, as variáveis utilizadas para a construção de modelos de *machine learning* foram diferentes daquelas dos escores TIMI e GRACE, o que impede qualquer extrapolação para o conceito “inteligência artificial *versus* estatística”. O único estudo que construiu os dois tipos de modelos a partir do mesmo conjunto de variáveis (tamanho da amostra 628 pacientes; 38 variáveis) não mostrou uma superioridade consistente de vários tipos de *machine learning* sobre a regressão logística tanto na capacidade discriminatória como na calibração.^14^ Além disso, uma revisão sistemática que avaliou 71 estudos que compararam o *machine learning* com a regressão logística não mostrou superioridade da primeira em relação à segunda abordagem.^15^ Assim, com base no conjunto de estudos em pacientes com dor torácica aguda, a superioridade ou não do *machine learning* ainda não foi esclarecida.

Nosso estudo indica que a inteligência artificial pode construir um modelo preciso a partir de uma amostra inferior a mil pacientes e dúzias de variáveis preditivas. Entretanto, ao contrário da atual onda da inteligência artificial, nós não encontramos uma superioridade em relação ao modelo de regressão logística. Nosso estudo reforça a estatística tradicional aplicada a um conjunto de dados que tenha suas premissas cumpridas. Resultados similares a favor da modelagem tradicional foram observados para a predição da piora de pacientes hospitalizados^16^ ou readmissão de pacientes com insuficiência cardíaca.^17^

Apesar de ambos os modelos terem preenchido os critérios de calibração, a regressão logística mostrou que o *machine learning* apresentou uma melhor calibração. Esse fato sugere que o *machine learning* possa necessitar de conjuntos de dados maiores para calibrar padrões e probabilidades.

Por outro lado, nossos resultados podem ser interpretados em favor do *machine learning*. Considerando que essa abordagem tem a capacidade de melhorar constantemente seu valor preditivo ao ser exposto a novos dados, começando de uma acurácia razoável no momento basal, o *machine learning* pode tornar-se um melhor modelo em longo prazo se exposto a dados administrativos contínuos. Essa hipótese precisa ser testada, mas o presente estudo apoia investir nessa possibilidade.

Deve-se ainda contextualizar a inteligência artificial em termos da tomada de decisão médica: ela não deve ser confundida com o conceito de certeza. O *machine learning* não deve ser uma mudança de paradigma na tomada de decisão, uma vez que ele tem o mesmo conceito de prover probabilidades de um desfecho, e não de certeza. Nesse sentido, a medicina continua a ser uma “ciência de incertezas e uma arte de probabilidade”, conforme William Osler definiu há várias décadas.^18^ Além disso, decisão não depende somente da predição de desfechos, mas também em seus efeitos negativos. Um desfecho altamente provável sem consequências sérias pode ser preferível que um desfecho pouco provável de consequências devastadoras. Assim, após avaliar probabilidade por meio de um modelo *machine learning*, o médico deve tomar uma decisão. Além do dano, o julgamento deve se basear no custo de se tentar prevenir o evento e possíveis consequências não intencionais. Portanto, o julgamento clínico não será substituído por modelos estatísticos ou algoritmos *machine learning*.

Acreditamos que nossa amostra cumpre premissas para se construir tanto um modelo estatístico como um modelo de inteligência artificial. O número de eventos foi grande o suficiente para o número de variáveis preditivas inseridas na regressão logística e análise discriminante. Porém, nossos dados foram para a análise de calibração, o número de eventos foi baixo em cada decil de probabilidade preditiva, o que torna imprecisa a estimativa das probabilidades observadas. Esse é nossa principal limitação.

## Conclusão

O presente estudo sugere que um modelo *machine learning* preciso, como instrumento de predição, pode ser gerado a partir de uma amostra relativamente simples, de tamanho moderado de pacientes. No entanto, o *machine learning* não se mostrou superior ao modelo estatístico de regressão logística.
