# Supplementary material for: Validação de um Algoritmo de Inteligência Artificial para a Predição Diagnóstica de Doença Coronariana: Comparação com um Modelo Estatístico Tradicional
Source: Arq Bras Cardiol. 2021 Nov 22;117(6):1061–70. [Article in Portuguese] doi: 10.36660/abc.20200302 (PMC8757162; doi:10.36660/abc.20200302)
Supplement: Translation

# Translation: Validation of an Artificial Intelligence Algorithm for Diagnostic Prediction of Coronary Disease: Comparison with a Traditional Statistical Model

**Authors:** Luis Correia, Daniel Lopes, João Vítor Porto, Yasmin F. Lacerda, Vitor C. A. Correia, Gabriela O. Bagano, Bruna S. B. Pontes, Milton Henrique Vitoria de Melo, Thomaz E. A. Silva, André C Meireles

**Affiliations:** 1 Salvador BA Brazil Escola Bahiana de Medicina e Saúde Pública, Salvador, BA – Brazil; 2 Salvador BA Brazil Hospital São Rafael, Salvador, BA – Brazil

**Keywords:** Validation Studies, Artificial Intelligence, Coronary Artery Disease/diagnostic, Data Interpretacion, Statistical

## Abstract

**Background:**

Multivariate prognostic analysis has been traditionally performed by regression models. However, many algorithms capable of translating an infinity of patterns into probabilities have emerged. The comparative accuracy of artificial intelligence and traditional statistical models has not been established in the medical field.

**Objective:**

To test the artificial intelligence as an accurate algorithm for predicting coronary disease in the scenario of acute chest pain and evaluate whether its performance is superior to traditional statistical model.

**Methods:**

A consecutive sample of 962 patients admitted with chest pain was analyzed. Two probabilistic models of coronary disease were built using the first two-thirds of patients: a machine learning algorithm and a traditional logistic model. The performance of these two predictive strategies were evaluated in the remaining third of patients. The final logistic regression model had significant variables only, at the 5% significance level.

**Results:**

The training sample had an average age of 59 ± 15 years, 58% males, and a 52% prevalence of coronary disease. The logistic model was composed of nine independent predictors. The machine learning algorithm was composed of all candidates for predictors. In the test sample, the area under the ROC curve for prediction of coronary disease was 0.81 (95% CI = 0.77 - 0.86) for the machine learning algorithm, similar to that obtained in logistic model (0.82; 95% CI = 0.77 - 0.87), p = 0.68.

**Conclusion:**

The present study suggests that an accurate machine learning prediction tool did not prove to be superior to the statistical model of logistic regression.

## Introduction

In the last decades, computers’ ability to generate and store data has improved substantially, leading to highly complex and large datasets. Traditional statistical modeling has the advantage of simplicity, as it fits the relationship between predictors and outcomes into a regression formula. However, these models have many assumptions that are difficult to be satisfied in complex sets of information: limited number of variables, adequate distribution, independence of observations, no multicollinearity, and concerns with interactions. In contrast, the prediction mechanism of artificial intelligence is algorithm-based, with no assumptions or limit of variables. Therefore, different from statistical modelling, prediction algorithms do not become less accurate as data get complex. In these scenarios of “big data”, artificial intelligence becomes more accurate than traditional statistics.^1,2^

Medical data can suffer from bias if not collected under a pre-established protocol. For this reason, the traditional epidemiological approach of small sets of data, prospectively collected, is the most appropriate choice in medical research.^3^ Therefore, it is important to explore whether artificial intelligence remains superior to statistical modelling if exposed to samples of moderate size and limited number of variables, as in most epidemiological studies.

Prediction of coronary artery disease (CAD) in patients with acute chest pain is a major challenge for the emergency physician who has to decide whether to discharge the patient, proceed with further non-invasive tests or go directly to invasive angiography. Discharging a patient with unstable coronary disease may be devastating, but admitting anybody with chest pain could have unintentional consequences.^4^ In this process, the probability of obstructive CAD should drive medical decision-making.^5^

In the present study, we utilized data from a prospective registry of chest pain^5^ to build a machine learning model to predict obstructive coronary disease. We aimed to evaluate whether an artificial intelligence algorithm is a better predictor than logistic regression in a traditional set of simple epidemiological data, considering both discrimination and calibration properties.

## Methods

### Sample selection

From September 2011 to November 2017, all patients admitted with chest pain and clinical suspicion of CAD (regardless of electrocardiogram or troponin results) to the coronary care unit of our hospital were included in the study. Exclusion criterion was patient’s refusal to participate. As defined *a priori*, a total of 962 patients were divided into the derivation sample (first two-thirds, n=641) and validation sample (last third, n= 321). The study was approved by an institutional review committee and that the subjects gave informed consent.

#### Predictors of obstructive CAD

At admission (baseline), three sets of variables were recorded as candidates for prediction of obstructive CAD. First, 13 variables related to medical history and clinical presentation; second, 14 characteristics of chest discomfort; third, 11 variables related to abnormalities in imaging or laboratory tests at admission: ischemic changes on electrocardiogram (T wave inversion ≥ 1 mm or dynamic ST deviation ≥ 0.5 mm), positive troponin (> 99^th^ percentile for the general population; Ortho-Clinical Diagnostics, Rochester, NY, USA), N-terminal pro-B-type natriuretic peptide (NT-proBNP, enzyme-linked fluorescent assay, Biomérieux, France), high-sensitivity C-reactive protein (CRP, nephelometry, Dade-Behring, USA), D-Dimer (immunoenzymatic essay, Biomérieux, France), low-density lipoprotein (LDL)-cholesterol (Friedwald equation), creatinine, white cell count, platelets, plasma glucose, and hemoglobin. Laboratory tests were performed in plasma material collected at presentation to the emergency department. Medical history and chest pain characteristics were recorded by three investigators (M.C., A.M.C., R.B.), trained to interview participants in a standardized manner to minimize bias and improve reproducibility. Radiologic signs of ventricular failure and the electrocardiogram were interpreted by the same investigator (L.C.).

### Outcomes

The primary outcome to be predicted by the model was diagnosis of obstructive CAD, defined by subsequent tests performed during hospital stay. Outcome data was collected by three investigators (M.C., A.M.C., R.B.) and confirmed by a fourth investigator (L.C.). For diagnostic evaluation, patients underwent invasive coronary angiography or a provocation test (perfusion magnetic resonance imaging, single-photon emission computed tomography or dobutamine stress echocardiogram), at the discretion of the assistant cardiologist. In case of a positive non-invasive test, patients had angiography for confirmation. Based on this diagnostic algorithm, obstructive CAD was defined as a stenosis ≥ 70% by angiography. A normal non-invasive test indicated absence of obstructive CAD and no further test was required. Regardless of coronary tests, patients were classified as “no obstructive CAD” if one of the following conditions was diagnosed by imaging test – pericarditis, pulmonary embolism, aortic dissection, or pneumonia.

### Statistical analysis

Shapiro-Wilk test was used to assess whether the data was normally distributed. For descriptive analysis, we used mean and standard deviation for continuous variables with normal distribution, and median and interquartile range for continuous variables without normal distribution. Category variables were described as frequencies. In the derivation sample, we first used unpaired Student’s *t* test for normally distributed continuous variables and Pearson’s chi-square test for univariate analysis of categorical variables. Numeric variables without normal distribution were analyzed by the non-parametric Mann-Whitney test. Then, variables with a p-value < 0.20 in the univariate analysis were included in the multivariate logistic regression analysis for prediction of obstructive CAD.

Multivariate models were developed by the stepwise method; all variables were fitted into a logistic regression model by using the forced entry and, at each step, the least significant stepwise term was removed from the model, using the Wald test. Initially, three intermediate models were built, according to the type of predictive variables (medical history, chest pain characteristics or physical exam/laboratory tests). Independent predictors (p < 0.10) in each intermediate model were included as covariates in the final model, constructed by including significant variables only, at the 5% significance level.

The machine learning algorithm recognizes patterns of clinical characteristics associated with outcome probabilities. Fisher discriminant analysis was used to generate dendrograms, which were combined repeatedly until the error ratio indicated optimal performance. The derivation sample was used for building the machine learning algorithm. Different from logistic regression, there was no preselection of variables and all 55 parameters were included with no further elimination. The influence of each variable on the probability calculation was defined by the purity of nodes and the percentage increase of associated error. As the result of the graphical analysis, we made 8,000 combination interactions.

The two models were compared in the validation sample. Area under the receiver operating characteristic (ROC) curves were used to test discrimination and compared between the models by DeLong's test. Calibration was evaluated by the Hosmer-Lemeshow test (applied to the probabilities generated by the models), and by calculating the calibration slope and intercept of the linear plot of mean predicted probability against observed incidence of events per deciles of prediction (a perfectly calibrated model has an intercept of 0 and slope of 1). Before performing linear regression, the following assumptions had to be verified: linear relationship, independence of observations, normality of residuals, homoscedasticity of residuals.

Statistical significance was defined as p < 0.05. The SPSS software was used for data analysis.

### Determination of sample size

Machine learning does not have sample size assumptions. For logistic regression, the derivation set was planned to allow inclusion of at least 15 covariates in logistic regression model. Calculation was based on the following assumptions: 50% prevalence of obstructive CAD and the need for 10 events for each covariate in the logistic regression model.^6,7^ Therefore, a minimum of 300 patients would be required in the derivation sample. The validation sample was set to test the discriminatory accuracy by the ROC curve analysis. Based on the assumption of an AUC of 0.70, to provide 90% power to reject the null hypothesis of AUC equal 0.50, with an alpha of 5%, a minimum of 85 patients was required. Therefore, a minimum of 100 patients would be required in the validation group. These assumptions were satisfied. The analysis of this sample was performed and completed in January 2018 to avoid multiple testing.

## Results

### Characteristics of the derivation sample

Six hundred forty-one patients were studied, aged 59 ± 15 years, 58% males, 30% with previous history of coronary disease. Median time elapsed between the onset of symptoms and first clinical evaluation in the hospital was 4.2 hours (interquartile range 1.9 - 14 hours). By using the study protocol, we identified 330 patients with obstructive CAD, a prevalence of 52%. All these cases had the diagnosis confirmed by invasive coronary angiography. Regarding the 311 patients without CAD, 93 were classified by a negative angiography, 169 by a negative noninvasive test and 52 had other dominant diagnosis (14 pulmonary embolism, five aortic dissection, 28 pericarditis, two pneumonia).

### Characteristics of the validation sample

Three hundred twenty-one patients were studied, with some characteristics similar to the derivation group, age of 59 ± 16 years, 58% males, 22% with previous history of coronary disease. Time elapsed between onset of symptoms and first clinical evaluation in the hospital had a median of 7.0 hours (interquartile range = 2.4 - 23 hours). Using the study protocol, we identified 163 patients with obstructive CAD, a prevalence of 51%. All these cases had the diagnosis confirmed by invasive coronary angiography. Regarding the 158 patients without CAD, 88 were classified by a negative angiography, 13 by a negative non-invasive test and 57 had another dominant diagnosis (25 pulmonary embolism, two aortic dissection, 25 pericarditis, five pneumonia).

### Development of the logistic model

Among the 13 variables related to medical history and clinical presentation, seven were positively associated with obstructive CAD at a significance level < 10%: age, male gender, acute left ventricular dysfunction, previous history of CAD, diabetes, smoking, and symptoms triggered by exercise – Table 1. When these seven variables were included in the logistic regression, previous history of CAD lost significance and all others remained significant at a level < 5% - (Intermediate Model 1, Table 2).

**Table 1:** Comparison of medical history, chest pain characteristics and laboratory tests between patients with and without obstructive coronary artery disease in the derivation sample

	Obstructive CAD		p-value
	Yes (N = 330)	No (N = 311)	
***Medical History***			
Age (years)	63 ± 13	56 ± 16	< 0.001
Male gender	226 (69%)	148 (48%)	< 0.001
Body Mass Index (Kg/m^2^)	28 ± 4.8	28 ± 5.9	0.86
Systolic Blood Pressure (mmHg)	154 ± 32	152 ± 30	0.55
Heart rate (bpm)	78.7 ± 19	79.4 ± 19	0.63
X-ray and clinical signs of LVF	41 (13%)	6 (2.0%)	< 0.001
History of CAD	113 (34%)	77 (25%)	0.01
Diabetes	122 (37%)	74 (24%)	< 0.001
Systemic hypertension	236 (72%)	210 (68%)	0.27
Current smoking	44 (13%)	26 (8.4%)	0.04
Family history of CAD	87 (26%)	79 (25%)	0.78
Exercise induction	50 (15%)	22 (7.1%)	0.001
Emotional induction	8 (2.4%)	15 (4.8%)	0.10
***Chest pain characteristics***			
Anterior left side location	268 (81%)	261 (84%)	0.37
Oppressive nature	189 (57%)	157 (51%)	0.09
Irradiation to neck	82 (25%)	74 (24%)	0.76
Irradiation to left arm	120 (36%)	93 (30%)	0.08
Vagal symptoms	146 (44%)	132 (42%)	0.65
Severe intensity	185 (56%)	150 (48%)	0.05
Number of episodes	1 (1 – 2)	1 (1 – 3)	0.14
Duration (minutes)	75 (20 – 129)	60 (11 – 214)	0.07
Intensity (1 – 10 scale)	7.7 ± 2.4	7.3 ± 2.4	0.03
Relief with nitrate	134 (41%)	98 (32%)	0.02
Similar to previous infarction	105 (32%)	76 (24%)	0.04
Worsening with compression	19 (5.8%)	43 (14%)	0.001
Worsening with position change	53 (16%)	60 (19%)	0.28
Worsening with arm movement	19 (5.8%)	31 (10%)	0.05
Worsening with deep breath	34 (10%)	68 (22%)	< 0.001
***Laboratory tests at admission***			
Ischemic changes on ECG	219 (66%)	119 (38%)	< 0.001
Positive troponin	215 (65%)	102 (33%)	< 0.001
NT-proBNP (pg/ml)	432 (155 – 1212)	73 (24 – 301)	< 0.001
Plasma creatinine (mg/dl)	0.90 (0.80 – 1.20)	0.80 (0.70 – 1.1)	< 0.001
LDL-cholesterol (mg/dl)	104 ± 53	108 ± 74	0.46
Plasma glucose (mg/dl)	130 (99 – 160)	107 (90 – 145)	0.009
C-reactive protein (mg/L)	7.4 (2.4 – 15)	6.3 (1.6 – 15)	0.003
White cell count	7.600 (6.050 – 10.100)	7.200 (5.700 – 9.550)	0.04
Platelets	232 (192 – 290)	232 (197 – 274)	0.83
D-Dimer (ng/ml)	474 (279 – 981)	424 (278 – 913)	0.43
Hemoglobin (g/dl)	14.1 ± 1.9	13.7 ± 1.7	0.11

CAD: coronary artery disease; Family history of CAD implies a first-degree female relative with disease before 55 years of age or first-degree male relative before 45 years of age; LVF: left ventricular failure; NT-pro-BNP: N-terminal pro b-type natriuretic peptide; ECG: electrocardiogram; LDL: low-density lipoprotein.

**Table 2:** Intermediate logistic regression models of medical history (Model 1), chest pain characteristics (Model 2) and laboratory tests (Model 3)

Variables	Multivariate significance level
***Model 1 (medical history)***	
Age (years)	< 0.001
Male gender	< 0.001
X-ray or clinical signs of LVF	< 0.001
Exercise trigger	0.005
Diabetes	0.009
Smoking	0.02
Previous CAD	0.32
***Model 2 (pain characteristics)***	
Worsening with deep breath	0.001
Worsening with compression	0.01
Severe intensity	0.01
Oppressive nature	0.06
Similar to previous infarction	0.08
Irradiation to left arm	0.16
Relief with nitrate	0.25
Duration (minutes)	0.32
Worsening with arm movement	0.67
***Model 3 (laboratory tests)***	
Ischemic changes on ECG	< 0.001
Positive troponin	< 0.001
NT-proBNP (pg/ml)	0.89
Plasma creatinine (mg/dl)	0.17
Plasma glucose (mg/dl)	0.12
C-reactive protein (mg/L)	0.58
White cell count	0.80

CAD: coronary artery disease; LVF: left ventricular failure; NT-pro-BNP: N-terminal pro b-type natriuretic peptide; ECG: electrocardiogram; LDL: low-density lipoprotein.

Regarding chest pain characteristics, among 14 variables, six had positive association with CAD: oppressive nature, irradiation to left arm, severe intensity, duration in minutes, relief with nitrates, similarity to previous infarction; and three had negative association with CAD: worsening with compression, arm movement and deep breath (Table 1). When these nine variables were added to the logistic regression, only three remained significant at a level < 5 – worsening with compression, deep breath and severe intensity (intermediate model 2, Table 2).

Among 11 laboratory tests, seven were positively associated with CAD: ischemic electrocardiogram, positive troponin, creatinine, glycaemia, NT-pro-BNP, CRP, white cell count (Table 1). When these seven variables were included in the logistic regression, only ischemic electrocardiogram and positive troponin remained significant at a level < 5% (intermediate model 3, Table 2).

The 11 significant variables in the intermediate model were included in the final logistic regression analysis, generating a final model with nine significant variables to predict the presence of CAD: age, male gender, ischemic electrocardiogram, positive troponin, left ventricular dysfunction, exercise induction, smoking, diabetes, and worsening with deep breath as the only “protective variable”. Regression coefficients and odds ratios are depicted in Table 3.

**Table 3:** Final model of logistic regression defining the independent predictors of obstructive coronary artery disease

Variables	βeta	Odds Ratio (95% IC)	p Value
Age (each year)	0.032	1.03 (1.02 – 1.05)	< 0.001
Male gender	1.04	2.8 (1.9 – 4.2)	< 0.001
Ischemic changes on ECG	1.05	3.0 (1.96 – 4.2)	< 0.001
Positive troponin	1.03	2.8 (1.9 – 4.1)	< 0.001
Signs of LVF	1.49	4.4 (1.7 – 12)	0.002
Exercise induction	0.93	2.5 (1.4 – 4.7)	0.003
Smoking	0.63	1.9 (1.5 – 3.4)	0.03
Diabetes	0.53	1.7 (1.1 – 2.6)	0.01
Worsening with deep breath	- 0.93	0.39 (0.23 – 0.68)	0.001
*Constant*	-3.70	----	----
***Excluded Variables***			
Severe Intensity	----	----	0.06
Worsening with compression	----	----	0.20

Hosmer-Lemeshow test = 4.1; p = 0.85; Area under the ROC curve of the model = 0.81; 95%CI = 0.77 – 0.84; p < 0.001. ECG: electrocardiogram; LVF: left ventricular failure.

### Development of the machine learning model

All 55 variables related to medical history, clinical presentation, chest pain characteristics and laboratory tests were included in the machine learning model. Performance of each variable in the model is depicted in Table 4 by the parameters of node purity and percentage increase in associated error.

**Table 4:** Model of machine learning showing the weight of each variable in defining probability, according to the parameters of nodes purity and percentage increase of associated error

	Parameters	
	Node purity	Error increase (%)
Age (years)	9.966665	0.015613620
Male gender	2.8464500	0.007500700
Weight (kg)	4.1309610	0.001209398
Height (cm)	3.4111841	0.001045826
Systolic blood pressure (mmHg)	4.9687120	0.001186313
Diastolic blood pressure (mmHg)	3.8970542	0.000573540
Heart rate (bpm)	4.8355910	0.001049536
X-ray and clinical signs of LVF	1.5479285	0.002145387
History of CAD	0.774541	0.000883823
History of angioplasty	0.809141	0.000852728
Past surgical revascularization	0.407289	0.000246474
History of stroke	0.502479	0.000155925
Carotid disease	0.352677	0.000111797
Peripheral artery disease	0.237674	0.000046758
Diabetes	0.606332	0.00041533
Systemic hypertension	0.680378	0.00059024
Current smoking	0.515775	0.00027025
Family history of CAD	0.471644	0.00002877
Statin therapy	0.496937	0.00023743
Aspirin therapy	1.004764	0.00120421
Chronic renal failure	0.137357	-0.000055424
Dialysis	0.016785	0.000007401
Menopause	0.683362	0.00094085
Hormone replacement therapy	0.379223	0.00010860
Physical/emotional trigger	1.951236	0.00097193
Anterior left side location	0.42644	0.00011250
Oppressive nature	0.90551	0.00070792
Irradiation to neck	0.41147	-0.00011320
Irradiation to left arm	0.70464	0.00025748
Vagal symptoms	0.493875	0.00003483
Severe intensity	0.624608	0.00016137
Intensity (0 – 10)	0.696121	0.00053586
Number of episodes	1.701348	0.00006361
Duration (minutes)	0.493875	0.00089453
Intensity (1 – 10 scale)	2.604802	0.00053586
Relief with nitrate	4.880035	0.00140420
Similar to previous infarction	0.696121	0.000699946
Worsening with compression	0.905519	0.000707922
Worsening with position change	0.384833	0.000041857
Worsening with arm movement	0.295489	-0.000075263
Worsening with deep breath	1.006767	0.000973174
Ischemic changes on ECG	4.880035	0.009409961
Positive troponin	7.935190	0.002336380
NT-proBNP (pg/ml)	17.39237	0.00367361
Plasma creatinine (mg/dL)	4.497093	0.00040330
Total cholesterol (mg/dL)	4.291174	0.00298651
LDL-cholesterol (mg/dL)	4.246389	0.00159658
HDL-cholesterol (mg/dL)	6.131821	0.00596194
Triglycerides (mg/dL)	5.213428	0.00397991
Plasma glucose (mg/dL)	4.115463	0.00222222
C-reactive protein (mg/L)	3.948830	0.00315613
D-dimer	3.418193	-0.00010837
White cell count	4.7122731	0.00034806
Hemoglobin (g/dL)	6.0717680	0.00230890
Platelets	5.0908595	0.00103027

CAD: coronary artery disease; LVF: left ventricular failure; NT-pro-BNP: N-terminal pro b-type natriuretic peptide; ECG: electrocardiogram; LDL: low-density lipoprotein; HDL: high density lipoprotein.

### Machine Learning versus Logistic Regression (validation sample)

Regarding discrimination, the area under the ROC curve of the machine learning probabilities was 0.81 (95% CI = 0.77 – 0.86), very similar to the area under the curve of logistic regression model 0.82 (95% CI = 0.78 – 0.87), p = 0.68 (Figure 1).

**Figure 2:** Area under the ROC curves of probabilities by the machine learning model and logistic regression model, respectively 0.81 (95% CI = 0.77 – 0.86) and 0.82 (95% CI = 0.78 – 0.87).

Regarding calibration, both models were validated by the Hosmer-Lemeshow test, but the logistic model showed lower level of significance of the difference between predicted and observed values (chi-square = 6.2; p = 0.62), as compared with the machine learning (chi-square = 12.9; p = 0.11), suggesting a better calibration of the first model.

Accordingly, linear regression between mean predictive probability and observed incidence of events per deciles of prediction showed an intercept of 0.010 (95% CI = -0.083 – 0.103) and slope of 1.004 (95% CI = 0.840 – 0.168) for logistic regression (r = 0.981). For machine learning, an intercept = -0.119 (95% CI = -0.296 – 0.059) and slope = 1.228 (95% CI = 0.909 – 1.547; r = 0.953) were found (Figure 2).

**Figure 1:** Scatter plot for linear regression analyses between mean predictive values per deciles and observed incidences. Panel A indicates calibration of machine learning model (intercept = -0.119, slope = 1.228, r = 0.953). Panel B shows calibration of logistic regression model (intercept = 0.010, slope = 1.004, r = 0.981).

## Discussion

In the present study, we tested the concept of building a machine learning tool for prediction of obstructive CAD in a small sample of patients with acute chest pain at admission, based on epidemiological data, prospectively collected, and a limited number of variables. First, we confirmed that artificial intelligence can be built from this type of data and be accurate in discrimination (yes or no) and calibration (probability prediction); second, our validation analysis suggested that artificial intelligence is not superior to traditional statistics in these circumstances.

In the fifties, the psychologist Paul Meehl demonstrated that statistical prediction is generally superior to clinical prediction by human judgement.^8^ This idea was supported by the work of Nobel laureate Daniel Kahneman, who described an array of cognitive bias responsible for inaccuracies of human heuristics.^9^ Such concepts supported the emphasis on using statistical models as the best evidence-based approach to diagnostic and prognostic predictions. More recently, artificial intelligence rouses as a more robust technique for building prediction tools.

Typically, artificial intelligence is derived from large databases, available from electronic records or web-based interfaces.^10^ It provides precision due to the enormous sample size and no assumptions regarding the number of variables, distribution, independence of observations, multicollinearity and concerns with interactions.^1^ However, since these large data sets are not collected for scientific purpose, they lack information quality.^3^ On the other hand, epidemiological prospective studies with planned, standardized, and *a priori* data collection, are the best method for generating data sets of ideal quality. In this circumstances, traditional statistical modellings usually have assumptions fulfilled and good performance. Thus, the question arises: in these ideal circumstances for statistical modeling, does artificial intelligence remain a superior technique?

In the scenario of acute coronary syndromes and traditional data sets, four authors have compared machine learning versus statistics. Three of the studies evaluated prognosis in acute coronary syndrome and compared machine learning with risk scores, showing some superiority in discrimination for artificial intelligence.^11-13^ However, in these studies, the variables used to build machine learning models were different from those of the TIMI and GRACE scores, which impairs any extrapolation for the concept of artificial intelligence versus statistics. The only study that built the two types of models from the same set of variables (sample size of 628; 38 variables) did not show consistent superiority of the several types of machine learning over logistic regression neither for discrimination nor calibration.^14^ Also, a systematic review that assessed 71 studies comparing machine learning and logistic regression, showed no superiority of the former over the latter.^15^ Therefore, based on the set of studies in patients with acute chest pain, whether machine learning is superior to traditional statistics is an unresolved issue.

Our study indicates that artificial intelligence can build an accurate model from a sample of less than a thousand patients and a few dozens of predictive variables. However, in contrast with the current hype about artificial intelligence, we did not find it superior to the logistic regression model. Our study reinforces traditional statistics applied to a data set that meet its assumptions. Similar results in favor of traditional modelling were observed for prediction of deterioration of hospitalized patients^16^ or readmission of heart failure patients.^17^

Despite both models fulfilled the calibration criteria, logistic regression showed a better calibration than machine learning. This suggests that machine learning might need larger data sets to calibrate patterns and probabilities.

On the other hand, our results may be interpreted in favor of machine learning. Considering that machine learning has the ability of constantly improve its predictive value as it is exposed to new data, starting with a reasonable accuracy at baseline, it might become a better model in the long run if exposed to continuous administrative data. Hypothesis that need to be tested, but the present study gives support to invest in this possibility.

One should also contextualize artificial intelligence in terms of medical decision making: it should not be confused with a concept of certainty. Machine learning will not be a paradigm shift in decision making, because if has the same concept of providing probabilities of an outcome, instead of certainty. In this sense, medicine continues to be the “science of uncertainty and art of probability”, as William Osler defined several decades ago.^18^ Furthermore, decision does not only depend on prediction of outcomes, but also on their negative effects. A highly probable outcome of no serious consequences might be preferable than a low probability outcome of devastating consequences. Thus, after assessing probability through a machine learning model, physician should exercise judgment. In addition to possible damage, judgment should be based on the cost of trying to prevent the event and possible unintended consequences. Thus, clinical judgement is not to be replaced by statistical models or machine learning algorithms.

We believe our sample meets assumptions for building both statistical and artificial intelligence models. Number of events was large enough for the number of predictive variables entered into the logistic regression and for discrimination analysis. However, for calibration analysis, the number of events was low in each decile of predictive probability, making estimation of observed probabilities imprecise. These are our main limitations.

## Conclusion

The present study suggests that an accurate machine learning prediction tool can be derived from a moderate size and relatively simple sample of patients. However, machine learning did not prove to be superior to the statistical model of logistic regression.
